# Dynamical effects on the magnetic properties of dithiazolyl bistable materials†
†Electronic supplementary information (ESI) available. See DOI: 10.1039/c4sc03930k



**DOI:** 10.1039/c4sc03930k

**Published:** 2015-01-23

**Authors:** Sergi Vela, Mercè Deumal, Motoyuki Shiga, Juan J. Novoa, Jordi Ribas-Arino

**Affiliations:** a Departament de Química Física and IQTCUB , Facultat de Química , Universitat de Barcelona , Av. Diagonal 645 , 08028-Barcelona , Spain . Email: jordi.ribas.jr@gmail.com ; Email: j.ribas@ub.edu; b Center for Computational Science and E-Systems , Japan Atomic Energy Agency , 148-4, Kashiwanoha Campus, 178-4 Wakashiba, Kashiwa , Chiba , 277-0871 , Japan

## Abstract

The magnetic properties of molecule-based magnets are commonly rationalized by considering only a single nuclear configuration of the system under study (usually an X-ray crystal structure). Here, by means of a computational study, we compare the results obtained using such a *static* approach with those obtained by explicitly accounting for thermal fluctuations, and uncover the serious limitations of the *static* perspective when dealing with magnetic crystals whose radicals undergo wide-amplitude motions. As a proof of concept, these limitations are illustrated for the magnetically bistable 1,3,5-trithia-2,4,6-triazapentalenyl (TTTA) material. For its high-temperature phase at 300 K, we show that nuclear dynamics induce large fluctuations in the magnetic exchange interactions (*J*_AB_) between spins (up to 1000% of the average value). These deviations result in a ∼20% difference between the 300 K magnetic susceptibility computed by explicitly considering the nuclear dynamics and that computed using the X-ray structure, the former being in better agreement with the experimental data. The unveiled strong coupling between *J*_AB_ interactions and intermolecular vibrations reveals that considering *J*_AB_ as a constant value at a given temperature (as always done in molecular magnetism) leads to a flawed description of the magnetism of TTTA. Instead, the physically relevant concept in this case is the statistical distribution of *J*_AB_ values. The discovery that a single X-ray structure is not adequate enough to interpret the magnetic properties of TTTA is also expected to be decisive in other organic magnets with dominant exchange interactions propagating through labile π–π networks.

## Abstract 2

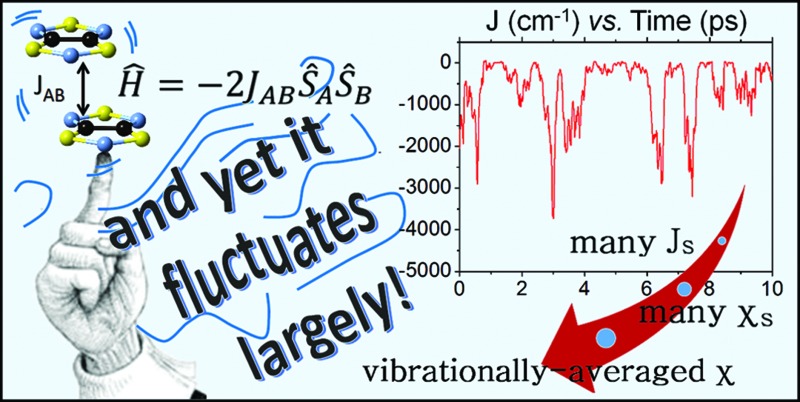
Using 1,3,5-trithia-2,4,6-triazapentalenyl material as a proof of concept, we demonstrate that vibrations of radicals can play a prime role in defining the magnetic properties of certain organic magnets.

## Introduction

The standard approach for the rationalization and accurate theoretical computation of magnetic properties in molecule-based systems draws on the assumption that these properties can be properly defined and determined by considering only a single nuclear configuration of the system under study (usually an X-ray recorded crystal structure). Within this approach, the magnetic exchange interactions between pairs of spin-carrying units (*J*_AB_) are evaluated at the fixed relative positions of these pairs within the crystal. Since the thermal oscillations of the spin carrying units around their equilibrium positions are ignored in this type of analysis, it can be stated that the standard approach is based on a *static* perspective. Note that such a *static* perspective also includes the increasingly acknowledged fact that *J*_AB_ values are significantly temperature-dependent in certain molecular materials due to thermal structural changes.^1–6^ Indeed, this temperature-dependence is commonly reported under the premise that the magnetic properties of the material at a given temperature can be properly evaluated using a single nuclear configuration at that temperature.

The *static* perspective employed in magnetism contrasts with the long-recognized need to account for thermal fluctuations in order to properly rationalize other physical properties (NMR/EPR parameters, absorption spectra, conductivity, *etc.*).^7–16^ Despite the study by Marx and coworkers on the dynamical magnetostructural properties of a [2Fe–2S] cluster embedded in a protein,^17^ the relevance of thermal fluctuations has not yet percolated in the field of molecule-based magnetic materials. Here, using the high temperature phase of the neutral radical 1,3,5-trithia-2,4,6-triazapentalenyl (TTTA) as a proof-of-concept system, we demonstrate for the first time the need to explicitly account for thermal vibrations in order to get a physically correct interpretation of the magnetic response of a molecular material. Due to the large-amplitude motions of the TTTA radicals in its high-temperature phase, the vibrationally-averaged structure obtained by diffraction measurements does not properly reflect all the configurations sampled due to thermal vibrations and, therefore, it is not sufficiently representative of the material. Consequently, the magnetism of the high-temperature phase of TTTA cannot be properly understood using the standard *static* perspective and one has to resort to a *dynamic* perspective, in which the nuclear motion is explicitly considered. We believe that the results obtained for TTTA will be relevant for molecule-based crystals whose spin carrying moieties undergo large-amplitude motions.

**Fig. 1:**
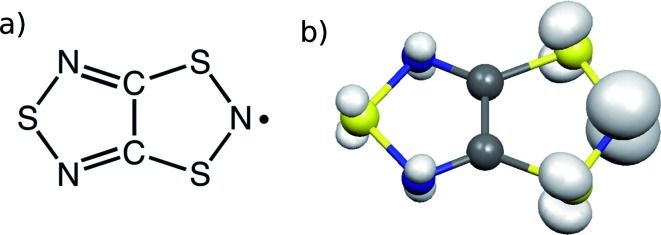
(a) TTTA chemical structure. (b) Spin density of a TTTA radical (cutoff at 0.007 a.u.).

**Fig. 2:**
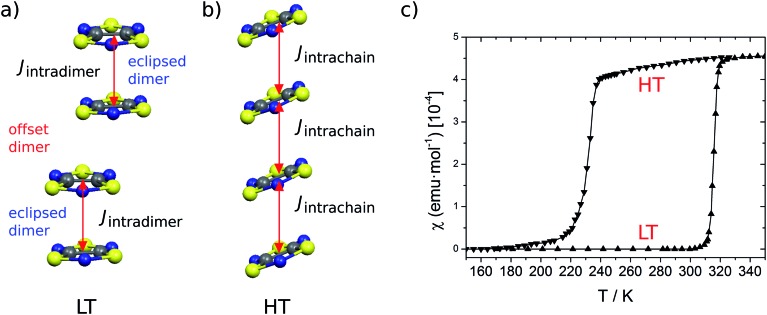
Lateral view of one stack of the X-ray structure at 300 K of both the LT (a) and HT (b) polymorphs of TTTA. The dominant magnetic interactions are marked in each stack. For the LT polymorph, the *J*_intradimer_ is shown between two eclipsed TTTA radicals, whereas *J*_AB_ is considered negligible for the slipped or offset pair. For the HT polymorph, the *J*_intrachain_ is shown within the 1D regular stack. (c) Temperature dependence of the magnetic susceptibility for TTTA on cooling (downward triangles) and on heating (upward triangles).

TTTA^18^ (Fig. 1) is one of a handful of molecule-based materials that exhibit bistability at room temperature. Its crystals undergo a first-order phase transition between their low-temperature (LT) diamagnetic and high-temperature (HT) paramagnetic phases, with a wide hysteresis loop encompassing room temperature (see Fig. 2). The columns of radicals present in the LT phase are distorted π-stacks comprising slipped pairs of nearly-eclipsed radicals (see Fig. 2a, S1, S3 and S4†). Conversely, the columns of the HT phase at room temperature are regular π-stacks of radicals, in which each molecule exhibits a slipped overlap with its two adjacent molecules along the stacking direction (see Fig. 2b, S2, S3 and S4†). In a previous computational work,^19^ we were able to rationalize the different magnetic response of the two phases of TTTA based on the common *static* perspective used in molecular magnetism. In particular, it was shown that the dominant *J*_AB_ interactions in the crystal structure of the LT phase at 300 K (LT-300) were those between the TTTA radicals forming eclipsed dimers. The corresponding large antiferromagnetic (AFM) interactions (*J*_intradimer_ = –1755 cm^–1^)^20^ were responsible for the overall diamagnetic behavior of this phase (see Fig. 2c). The dominant *J*_AB_ couplings in the crystal structure of the HT phase at 300 K (HT-300), in turn, were found to be the interactions between adjacent radicals within the regular stacks, which gave rise to a magnetic topology consisting of regular 1D AFM chains. The moderate *J*_AB_ value of these chains (at 300 K, *J*_intrachain_ = –135 cm^–1^) explained the “quenched” paramagnetism observed in experiments (see Fig. 2c).

**Fig. 3:**
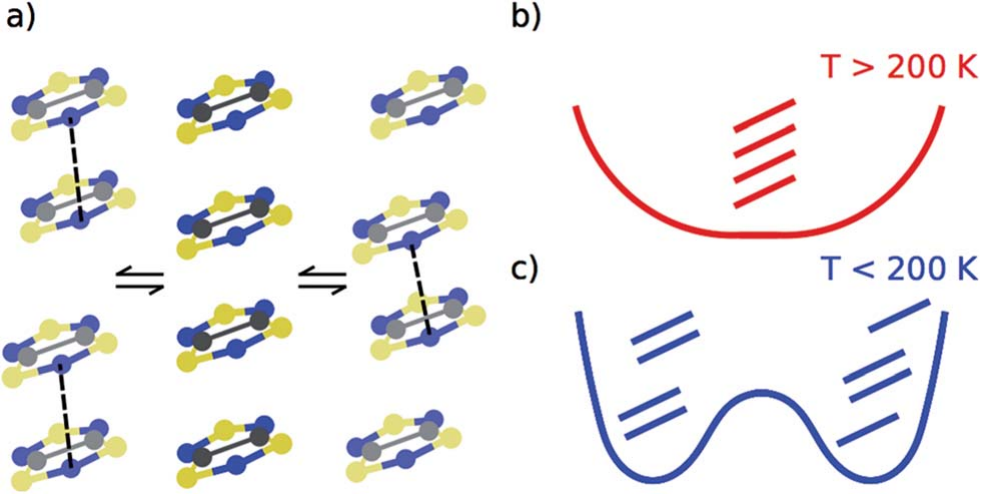
(a) The regular π-stacks of the HT polymorph (center) result from the dynamic interconversion between two distorted stacks (left- and right-hand side). (b and c) Schemes showing the temperature-dependence of the free energy profile of one column of four TTTA radicals with respect to an intrastack dimerization process. The three TTTA arrangements are a schematic representation of 3a.

Despite our previous computational work^19^ and the large number of studies devoted to the prototypical bistable TTTA material over the last years,^21–31^ it was not until recently that it was uncovered that each regular stack of the HT phase of TTTA is the resulting average structure of a unique fast intrastack pair-exchange dynamics, which is characterized by a rapid interconversion between the two distorted stacks displayed in Fig. 3a.^32^ Along the motions associated with this pair-exchange dynamics, a given TTTA radical continually exchanges the adjacent TTTA neighbor (upper or lower) with which it forms an eclipsed dimer. Although the regular stacking motif is not a minimum in the potential energy surface (PES) of the system, it is a minimum in the free energy surface (FES) at room temperature (Fig. 3b).^32^ Upon cooling, the pair-exchange dynamics gradually slows down and, at a temperature around 200 K, the stacks of the HT polymorph undergo a second order (or order–disorder) phase transition, by virtue of which the regular stack associated with a minimum at 300 K transforms into a transition state connecting two different new minima, each of them associated with a distorted or dimerized stack (Fig. 3c). The stacks of the LT polymorph, by contrast, remain in a dimerized (or ordered) state over the whole range of temperatures for which this polymorph has been observed (*T* < 310 K). It thus follows that the eclipsed TTTA dimers in the LT polymorph at room temperature are preserved despite the thermal fluctuations because TTTA radicals in this polymorph do not feature any pair-exchange dynamics.^32^

The discovery of the dynamic disorder concomitant to the pair-exchange dynamics of the HT phase of TTTA prompted us to explore the impact of the large-amplitude fluctuations undergone by the TTTA radicals on the microscopic *J*_AB_ interactions and on the macroscopic magnetic susceptibility (*χ*) of the material. Herein, by means of a computational study, we show that the vibrationally-averaged magnetic susceptibility (*χ̄*_vib_, *i.e.*, the magnetic susceptibility averaged over all nuclear configurations sampled due to thermal fluctuations) of the HT phase of TTTA at 300 K is substantially different than the magnetic susceptibility obtained using the X-ray structure as a single *static* configuration. This originates in the large fluctuations of the *J*_AB_ interactions between adjacent radicals as they oscillate around their equilibrium positions. Our results thus show that intermolecular vibrations exert a notable impact on the magnetic behavior of TTTA.

At this point, it should be stressed that our objective has not been to calculate the whole *χ*(*T*) curve of the HT phase, but to demonstrate that the *static* perspective does not necessarily provide all the insight required for an adequate interpretation of the magnetic properties of certain molecule-based materials. The computation of *χ̄*_vib_ at 300 K, which already entailed a major computational effort, suffices to prove this concept. The key role of intermolecular vibrations in defining the magnetic properties of materials, herein demonstrated for TTTA, will likely be a concept to be reckoned with when analyzing the magnetism of other members of the family of switchable dithiazolyl-based materials^33–40^ and, possibly, of other purely organic magnets^41–45^ (including other families of organic materials undergoing spin transitions^46–51^). Besides magnetism, we believe that thermal fluctuations will also be important for interpreting other physical properties (such as non-linear optics and conductivity) of molecular crystals based on neutral radicals.^52–55^

## Methodological details

The computational scheme that we adopted for the study of the interplay between thermal fluctuations and magnetism in TTTA consists of three steps: (i) we first ran *ab initio* molecular dynamics (AIMD) simulations^56,57^ at 300 K for both the LT and HT phases of TTTA; (ii) we then computed the *J*_AB_ values between pairs of radicals for a large number of frames along the AIMD trajectories; and in the last step, (iii) we calculated *χ̄*_vib_ on the basis of full diagonalizations of the Heisenberg Hamiltonian built from the previously evaluated *J*_AB_ values. In what follows, these three steps will be described in more detail.

### 1. AIMD simulations

As described in our previous work on the structure and dynamics of the two polymorphs of TTTA,^32^ the AIMD simulations at 300 K for the LT and HT polymorphs of TTTA were carried out using a triclinic supercell and a monoclinic supercell, respectively (see Fig. S5 and S6 and Table S1†). Both HT and LT supercells include 8 stacks of radicals, each of them containing 4 radicals (that is, a total of 32 TTTA molecules). These AIMD simulations for both polymorphs were run for *ca.* 10 ps and were performed using plane wave pseudopotential DFT^58^ calculations and the efficient Car–Parrinello propagation scheme,^57^ as implemented in the CPMD package.^59^ These calculations were carried out using the PBE exchange-correlation functional^60^ within the spin unrestricted formalism (broken symmetry singlet *M*_S_ = 0 state), together with Vanderbilt ultrasoft pseudopotentials,^61^ and *Γ*-point sampling of the Brillouin zone. The plane wave basis set was expanded at a kinetic energy cutoff of 25 Ry. The van der Waals interactions between the TTTA molecules were properly taken into account by adding the semiempirical dispersion potential introduced by Grimme,^62^ in its DFT-D2 parameterization, to the conventional Kohn–Sham DFT energy. The molecular dynamics time step was set to 4 a.u. and the fictitious mass for the orbitals was chosen to be 400 a.u. The AIMD simulations were performed in the canonical (or NVT) ensemble using Nosé–Hoover chain thermostats^63^ in order to control the kinetic energy of the nuclei and the fictitious kinetic energy of the orbitals. The temperature of the nuclei was set to 300 K. Periodic boundary conditions in all three directions were imposed in the simulations.

Concerning the use of PBE-D2 in the AIMD simulations, it should be mentioned that a series of recent benchmark calculations have shown that the use of PBE together with the Grimme correction furnishes good predictions for the structure and cohesive energies of molecular crystals in which closed shell molecules are held together by weak intermolecular forces.^64^ Even though radical···radical interactions were not included in Grimme's parameterization set,^62^ a most recent benchmark study^65^ has demonstrated that PBE-D2 provides excellent equilibrium distances and good interaction energies for π-dimers of radical ions presenting long, multicenter bonds (alternatively called pancake bonds), like those found in TTTA dimers. In fact, PBE-D2 has already been shown to provide a difference in cohesive energies between the two polymorphs of TTTA that is in good accordance with the experimental data.^32^ This good agreement, together with other validation studies included in ref. 32, demonstrates that PBE-D2 furnishes a correct description of the intermolecular interactions between TTTA radicals.

### 2. Evaluation of the magnetic coupling interactions

For every calculation of a *J*_AB_ value along the AIMD trajectory, the molecular configuration of the corresponding pair of TTTA radicals was excised from the supercell of 32 radicals. Since the evaluation of the time-evolution of the *J*_AB_ interactions between all pairs of radicals contained in the supercells would be too demanding in terms of computational cost, only a specific subset of pairs of radicals was considered.

**Fig. 4:**
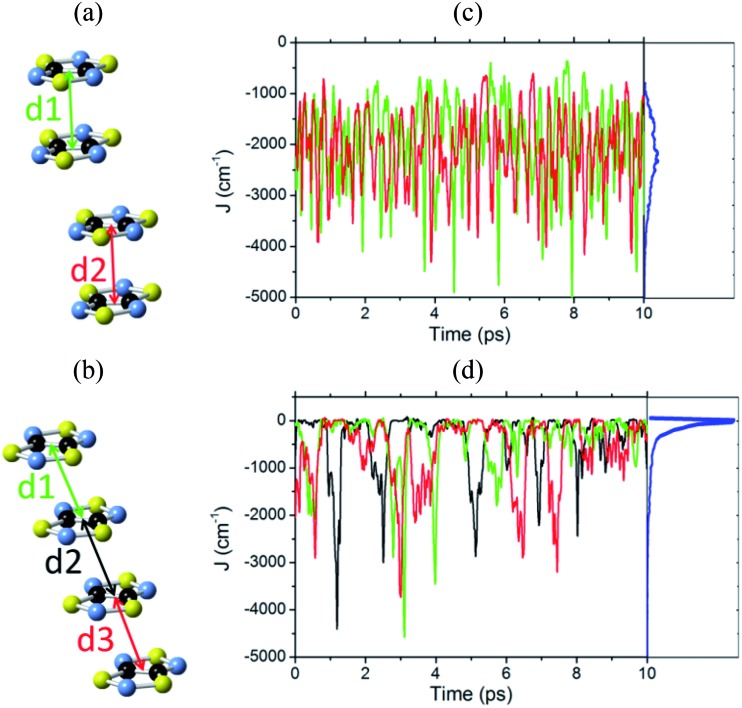
One stack of radicals of the supercell employed in the AIMD simulations of (a) LT-300 and (b) HT-300, which involves two/three different *J*_AB_ values between adjacent radicals. Time-resolved fluctuations of (c) *J*_1_ and *J*_2_ for the LT polymorph, and (d) *J*_1_, *J*_2_ and *J*_3_ for the HT polymorph. Each of these *J*_*n*_ values corresponds to the pair of radicals marked with *d*_*n*_ in (a) and (b). The blue curve of the right-most graphic is the probability distribution function (PDF) of the *J*_AB_ values, obtained by taking into account all the sampled values.

For the LT phase of TTTA, the evaluation of the time-evolution of magnetic coupling interactions was performed for the two eclipsed dimers within one of the stacks of the LT supercell. In other words, we inspected the time-evolution of two different *J*_AB_ interactions (see Fig. 4a). The values of these interactions were computed for molecular configurations sampled every 0.97 fs; overall, more than 20 000 *J*_AB_ evaluations were carried out for the LT polymorph. The magnetic exchange interaction associated with the central slipped pair of the distorted stacks of the LT polymorph (see Fig. 4a) was not considered in this study because it was previously demonstrated^19^ that this interaction is negligible compared to the exchange interactions of the eclipsed dimers. The exchange interactions between radicals belonging to different stacks were not considered either for the same reason.

For the HT phase of TTTA, the evaluation of the time-evolution of the *J*_AB_ values was carried out for all the nearest-neighbor radical pairs within two of the stacks of the HT supercell. Since each stack comprises four different TTTA radicals and, thus, three different pairs (see Fig. 4b and S7†), it follows that we inspected the time-evolution for six different radical pairs of the HT phase. The value for these six different *J*_AB_ interactions was computed for molecular configurations sampled every 0.97 fs; overall, more than 60 000 *J*_AB_ evaluations were carried out for the HT phase. Besides, we also inspected the time-evolution of the most relevant exchange interactions between radicals belonging to different stacks (see Fig. S8†).

Let us now explain how the *J*_AB_ values were evaluated. From the general Heisenberg Hamiltonian for a pair of *S* = 1/2 spin centers,

1 *Ĥ* = –2*J*_AB_*ŝ*_A_*ŝ*_B_

the *J*_AB_ value is defined as 2*J*_AB_ = *E* ^S^ – *E* ^T^, where *E* ^S^ and *E* ^T^ are the energies of the singlet and triplet states, respectively, of a two-TTTA radical cluster. In DFT calculations, the energy of the singlet state can be approximated using that of the single-determinant broken-symmetry (BS) solution.^66^ Within this approximation, the expression chosen to compute the energy difference is^67^
2
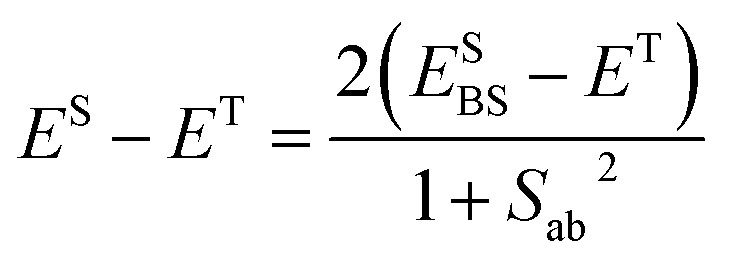
where *E*SBS is the energy of the BS solution and *S*_ab_ is the overlap integral between the magnetic α and β orbitals of the BS solution. In our case, those orbitals are localized on each of the two radicals. This leads to *S*_ab_ ≈ 0 and to the final expression that was used to compute *J*_AB_ values:

3 *J*_AB_ = *E*SBS – *E*^T^

Both *E*SBS and *E* ^T^ were computed at the UB3LYP^68^/6-31+G(d)^69^ level as implemented in Gaussian 03.^70^ It should be noted that the use of eqn (3) is an approximation that might entail a certain error that is difficult to control. However, the results collected in Table S2† prove that this equation (in combination with UB3LYP/6-31+G(d) calculations) provides results that compare reasonably well with those obtained with correlated wavefunction methods. It thus follows that the way chosen to compute *J*_AB_ values offers a good compromise between accuracy and computational efficiency.

### 3. Evaluation of the vibrationally-averaged magnetic susceptibility

The vibrationally-averaged magnetic susceptibility *χ̄*_vib_ for the HT phase at 300 K was computed by averaging the *χ* value over the whole set of configurations that were used to determine the time-evolution of the *J*_AB_ values between adjacent radicals within a stack. That is to say, the *χ* value was computed for an overall of *ca.* 10 000 different molecular configurations (each configuration was collected every 0.97 fs throughout the AIMD simulations). Given a frame along the AIMD trajectory of the HT phase and the sequence of *J*_AB_ values associated with the molecular configurations of the two stacks herein considered, the magnetic susceptibility was computed by means of a full diagonalization of the matrix representation of the following Heisenberg Hamiltonians:

4 *Ĥ*_1_ = –2*J*_1_*ŝ*_1_*ŝ*_2_ – 2*J*_2_*ŝ*_2_*ŝ*_3_ – 2*J*_3_*ŝ*_3_*ŝ*_4_ (for the first stack)

5 *Ĥ*_2_ = –2*J*_4_*ŝ*_5_*ŝ*_6_ – 2*J*_5_*ŝ*_6_*ŝ*_7_ – 2*J*_6_*ŝ*_7_*ŝ*_8_ (for the second stack)

where *J*_1_, *J*_2_ and *J*_3_ refer to the magnetic coupling interactions associated with the *d*_1_, *d*_2_ and *d*_3_ pairs of radicals of Fig. 4b, and *J*_4_, *J*_5_ and *J*_6_ refer to the magnetic coupling associated with the *d*_4_, *d*_5_ and *d*_6_ pairs of radicals of Fig. S7.† Note that each of these Hamiltonians corresponds to a 1D magnetic model system with 4 spin centers. The intercolumn *J*_AB_ values were not taken into account when diagonalizing the Hamiltonians of eqn (4) and (5) because the interstack *J*_AB_ values that are sampled along the AIMD trajectory are much smaller than the corresponding intrastack values (see results and discussion subsection 1). The diagonalization of the above Hamiltonians (whose matrix representation has a dimension of 6 by 6) furnishes the energy levels and associated spin quantum numbers for every sequence of *J*_AB_ values within a stack that has been sampled during the AIMD simulations. With this energy spectrum, the value of *χ* for each stack can be straightforwardly evaluated using standard statistical mechanics expressions. The vibrationally-averaged magnetic susceptibility reported in this article corresponds to the average of the *χ* values computed for *Ĥ*_1_ and *Ĥ*_2_ along the AIMD trajectory.

In order to assess the importance of thermal fluctuations, the vibrationally-averaged magnetic susceptibility computed at 300 K was compared to the experimental value and to the *static* magnetic susceptibility associated with the “frozen” X-ray crystal structure of the HT phase at 300 K. Such *static* magnetic susceptibility was obtained upon diagonalization of the Heisenberg Hamiltonian of eqn (1) with *J*_1_ = *J*_2_ = *J*_3_ = –135 cm^–1^.^19^ Note that this value corresponds to the *J*_AB_ value between two adjacent radicals within a stack for the regular HT structure refined at 300 K. In other words, the *static* magnetic susceptibility was obtained following our First-Principles Bottom-Up (FPBU) approach,^71^ which has been successfully used over the last years to rationalize the magnetic properties of multiple molecule-based materials.^72^

## Results and discussion

### 1. Impact of the thermal intermolecular vibrations on the *J*_AB_ values

Before presenting the fluctuations featured by the exchange interactions between neighboring radicals, it should be mentioned that the computed average structures and the computed thermal ellipsoids for both the LT and HT polymorphs of TTTA are in good agreement with the experimental data. This confirms the quality of the AIMD trajectories obtained (see Fig. S9 and S10†). In fact, the trajectories employed for the evaluation of the time-evolution of the *J*_AB_ values were also employed in ref. 32 for the inspection of the dynamics of both polymorphs of TTTA.

The time-resolved evolution of the *J*_AB_ values for the stacks of LT-300 and HT-300 are displayed in Fig. 4c and d, respectively. It is observed that the *J*_AB_ values of both polymorphs feature remarkable large-amplitude fluctuations. In the LT polymorph, these fluctuations span a broad range of values that are restricted to the strong AFM region (from *ca.* –400 cm^–1^ to –5000 cm^–1^). These fluctuations resemble those of two independent harmonic oscillators because the vibrations of TTTA radicals around their equilibrium positions in the LT polymorph at 300 K are to a large extent harmonic. In other words, the *J*_AB_ fluctuations in the LT polymorph reflect that the eclipsed dimers of this phase are preserved during the AIMD simulations. The probability distribution function (PDF) associated with the *J*_AB_ values that are sampled along the AIMD trajectory of the LT polymorph is locally quite flattened around the maximum (found at about –2500 cm^–1^; see blue curve in Fig. 4c). The resulting average value of this PDF is 
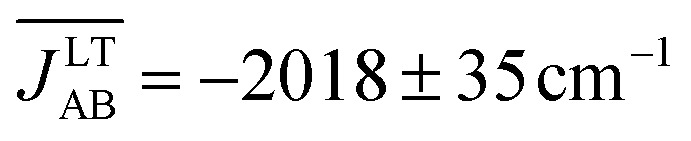
 (see Table S3†), which is *ca.* 15% more AFM than the corresponding X-ray crystal *J*LT,X-rayAB value (*i.e.*, the *static* value extracted from a single point energy calculation at the X-ray structure: –1755 cm^–1^).^19^

**Fig. 5:**
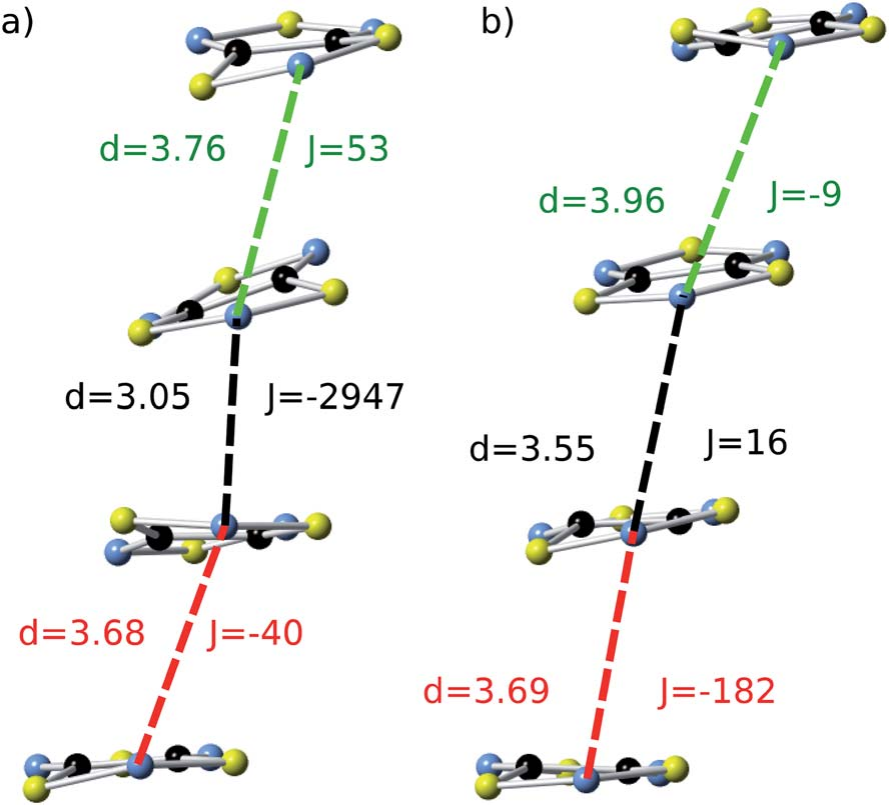
Two representative snapshots of the configurations associated with the time-resolved fluctuations of the *J*_AB_ values of the HT polymorph (Fig. 4d). The snapshot shown in (a) corresponds to a configuration collected at *t* = 2.5 ps, for which the central radicals form a quasi-eclipsed dimer. The snapshot displayed in (b) corresponds to a configuration collected at *t* = 4.5 ps, for which all the adjacent radicals exhibit a slipped overlap. For each pair of adjacent radicals, two different values are given: (i) the distance (*d*, in Å) between the nitrogen atoms of the S–N–S moieties of the two radicals, and (ii) the corresponding *J*_AB_ value (*J*, in cm^–1^). Note that the color code used to identify each pair within the stack is the same as the one employed in Fig. 4b and 4d.

The time-resolved evolution of the *J*_AB_ values in the HT polymorph markedly differs from the fluctuations expected for a set of harmonic oscillators. In Fig. 4d, it can be observed that there are time intervals in which some of the *J*_AB_ values are strongly AFM while others are close to zero (either weakly AFM or FM), and time intervals in which all the *J*_AB_ values of one stack adopt weak AFM values or even weak ferromagnetic (FM) values. The former type of time intervals is associated with configurations in which the presence of eclipsed dimers gives rise to strong AFM *J*_AB_ values (Fig. 5a). Contrarily, the latter type of time intervals includes configurations that look similar to the regular stacking motif observed in X-ray measurements (*i.e.*, configurations in which all the adjacent radicals exhibit a slipped overlap, Fig. 5b). It thus follows that the anharmonic fluctuations of the *J*_AB_ values in the HT polymorph at 300 K reflect the pair-exchange dynamics taking place within its stacks. The range of *J*_AB_ values sampled in the HT polymorph (see also Fig. S7†) is wider than that of the LT polymorph: from slightly positive values (moderate ferromagnetic FM interactions, *ca.* 70 cm^–1^) to strongly AFM values, some of them being as large as –5000 cm^–1^. The PDF associated with the *J*_AB_ values that are sampled along the AIMD trajectory of the HT polymorph is completely different from that of the LT polymorph because it features a non-Gaussian shape with a pronounced peak at about 0 cm^–1^ (see blue curve in Fig. 4d). The resulting average of this PDF is 
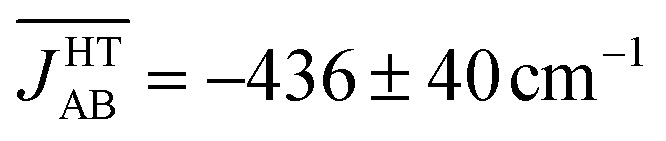
 (see Table S3†), which is much more AFM (*ca.* 200%) than both the most probable value of the PDF (*ca.* 0 cm^–1^) in Fig. 4d and the value extracted from the X-ray crystal structure (*J*HT,X-rayAB = –135 cm^–1^). The unexpectedly large value of 
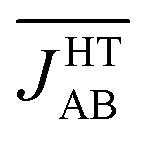
 is due to the strongly AFM values that are occasionally sampled during the time-evolution of the *J*_AB_ interactions of this polymorph (Fig. 4d).

With reference to the interstack *J*_AB_ values of the HT polymorph, Fig. S8† shows that the amplitude of their fluctuations is much smaller (<1%) than that of the intrastack *J*_AB_ values. Furthermore, most of the values sampled by the interstack *J*_AB_ values along the AIMD trajectory are close to zero. It thus follows that these *J*_AB_ interactions do not play any relevant role in defining the magnetic response of the HT polymorph. This is in line with our previous work on the magnetic properties of TTTA within a *static* perspective,^19^ where it was demonstrated that the magnetic coupling interactions between radicals belonging to different stacks are dwarfed by the intrastack exchange interactions. For these reasons, the interstack *J*_AB_ interactions were not taken into account when computing *χ̄*_vib_.

### 2. Statistical magneto-structural correlations

**Fig. 6:**
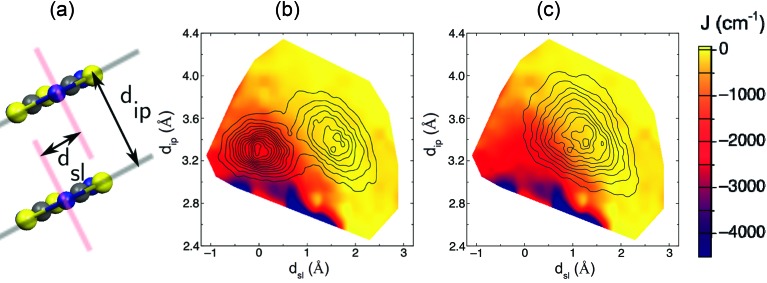
(a) Variables *d*_ip_ and *d*_sl_ used to analyze the magnetostructural correlations present in TTTA. Combination of the 2D-PDF of the values sampled for these two structural variables along the AIMD trajectory (black contours) with the associated *J*_AB_ value (colored surface) at each point in the (*d*_ip_, *d*_sl_) subspace, for both the (b) LT and (c) HT polymorphs at 300 K. Note that the left-most (right-most) peak in the bimodal distribution of LT corresponds to the eclipsed (offset) dimers of the distorted stacks of the LT polymorph. These PDFs were obtained from the configurations sampled during the AIMD simulations at 300 K. For the sake of clarity, the contours have been capped at a 0.1% probability, which means that there is a region outside the external contour that has non-zero probability of being explored. In particular, the short-lasting events in which the *J*_AB_ interaction between a given radical pair reaches up to –5000 cm^–1^ are not represented in this figure since they are rare in terms of statistics.

As explained in the previous subsection, the PDFs associated with the *J*_AB_ values that are sampled along the AIMD trajectories at 300 K for the LT and HT polymorphs of TTTA are markedly different. In the following, we will clarify the reasons behind this observation by means of a statistical magneto-structural correlation analysis that takes into account the molecular configurations and the *J*_AB_ values sampled along the AIMD simulations. The two structural variables chosen for this study are *d*_ip_, which measures the interplanar distance between adjacent radicals in one stack, and *d*_sl_, which measures the degree of relative slippage between adjacent radicals within a stack (see Fig. 6a). The strength of the magnetic exchange coupling for each possible combination of the two structural variables was obtained by averaging all the computed *J*_AB_ values of the configurations that present a given set of *d*_ip_ and *d*_sl_ along the AIMD trajectory. The colored surfaces in Fig. 6b and c show the dependence of *J*_AB_ on *d*_ip_ and *d*_sl_. At this point, it is important to stress that these surfaces are a fingerprint of TTTA and, thus, the magnetic properties of a given phase of this material at a given temperature depend on which *J*_AB_ values are sampled due to the thermal fluctuations of the radicals.

For the LT phase, the two-dimensional PDFs associated with the sampled values of *d*_ip_ and *d*_sl_ (Fig. 6b) present two peaks that correspond to the eclipsed dimer (left-most peak) and to the slipped or offset pair (right-most peak), which are the two classes of dimers within a column of radicals, as depicted in Fig. 2. As previously mentioned, the slipped pair explores a region associated with very small *J*_AB_ values, in contrast to what is observed for the eclipsed dimers (Fig. 6b). Indeed, the thermal motion of the eclipsed dimer is contained in the region of small values of *d*_sl_, which are associated with strongly antiferromagnetic *J*_AB_ values (Fig. 6b, –1 < *d*_sl_ < 1). This explains the flattened distribution of magnetic exchange couplings centered *ca.* –2000 cm^–1^ observed in Fig. 4c. In turn, the dimers of the HT polymorph feature oscillations of a larger amplitude than those of the LT polymorph, occasionally reaching the strong AFM region. However, their thermal motion is centered on the configuration observed in the X-ray structure of the HT polymorph at 300 K (*d*_sl_ = 1.3, *d*_ip_ = 3.4), whose associated *J*_AB_ value is –135 cm^–1^, and on the surrounding area associated with very weak AFM (or even weak FM) *J*_AB_ values (Fig. 6c). As a result, since the *J*_AB_ surface is rather flat on this region, the corresponding PDF of the sampled *J*_AB_ values features a maximum in the region of very weak AFM *J*_AB_ values (Fig. 4d).

**Fig. 7:**
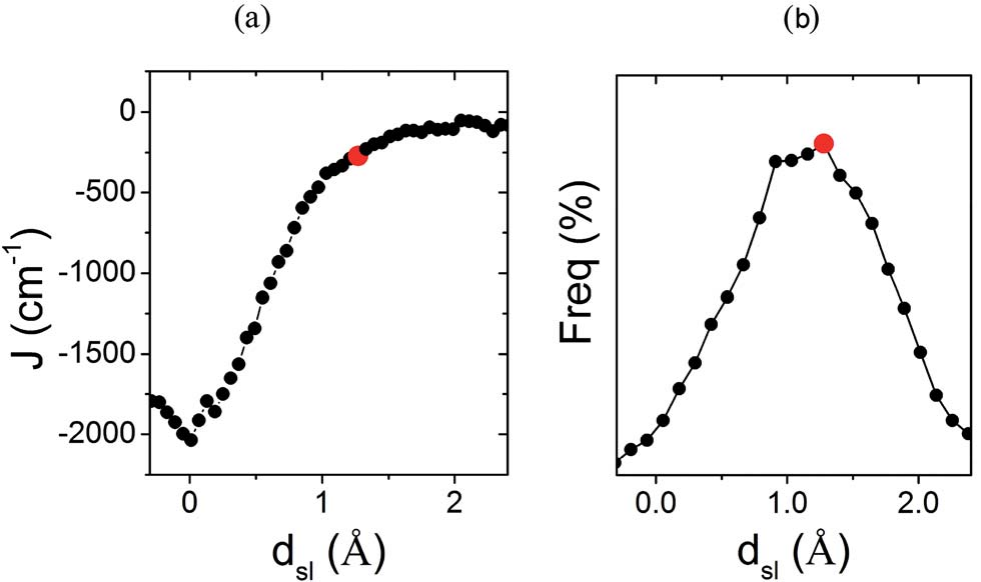
(a) Dependence of the *J*_AB_ value between adjacent radicals on the degree of relative slippage (*d*_sl_) and (b) the probability distribution function (PDF) associated with *d*_sl_. Each *J*_AB_ value on (a) has been computed as an average of all the computed *J*_AB_ values for all the configurations sampled for a given value of *d*_sl_. The red mark indicates the value of *d*_sl_ for the HT polymorph X-ray structure (*d*X-raysl). Note that *d*_sl_ is defined in Fig. 6.

The previous analysis provides a rationale for the different distribution of *J*_AB_ values sampled for the LT and HT polymorphs at 300 K, but does not explain why 
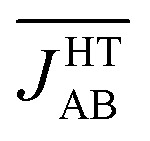
 is much more antiferromagnetic than *J*HT,X-rayAB. In order to understand this result, it is better to explore the magneto-structural correlations by considering the two structural variables separately from each other. It can be observed in Fig. 7a that the evolution of *J*_AB_ features an exponential dependence with respect to a change in the *d*_sl_ parameter, within the range of *d*_sl_ values that are sampled due to intermolecular vibrations (see Fig. S11† for the dependence of *J*_AB_ on *d*_ip_). Due to this exponential dependence, the variation of *J*_AB_ along *d*_sl_ is largely asymmetric with respect to a change in its value from the average geometry (*i.e.* the X-ray geometry). For instance, relatively small distortions towards smaller values of *d*_sl_ give rise to a large change of *J*_AB_, by virtue of which this quantity becomes exceedingly antiferromagnetic. This is because the eclipsed configurations imply a better overlap between the SOMOs of the TTTA radicals. In contrast, a distortion in the opposite sense, that is, towards a larger *d*_sl_ value, produces a much softer change in *J*_AB_. Given that the PDF of the *d*_sl_ values sampled along the AIMD trajectory is a quasi-normal distribution centered in the X-ray value (Fig. 7b), the occurrence of negative displacements (*d*_sl_ < *d*X-raysl) is nearly the same as the one for positive displacements (*d*X-raysl < *d*_sl_). However, the effect of the negative displacements on the magnetic exchange interaction is much more important. Thus, it can be concluded that the difference between 
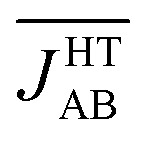
 and *J*HT,X-rayAB arises from the asymmetric response of *J*_AB_ with respect to the geometrical changes caused by the large amplitude thermal fluctuations. At this point, it should be mentioned that similar exponential variations of *J*_AB_ with respect to *d*_ip_ and *d*_sl_ have been reported for other radicals.^73–75^

### 3. Impact of the thermal intermolecular vibrations on the magnetic susceptibility

We will now explore how the notable impact of thermal fluctuations on the microscopic *J*_AB_ values manifests itself on a macroscopic property. At first glance, the value obtained for 
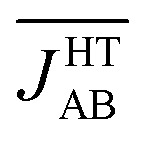
 would seem to be incompatible with the experimental magnetic response of the HT phase. Indeed, a 1D regular AFM chain with a *J*_AB_ value of –436 cm^–1^ would result in a vanishingly small value of magnetic susceptibility (*χ* = 1.4 × 10^–4^ emu mol^–1^), which is at odds with the observed paramagnetic behavior (the experimental susceptibility at 300 K^18^ is *χ* = 4.5 × 10^–4^ emu mol^–1^, see Table 1). Therefore, the premise that the time-average of the magnetic coupling interactions defines the magnetic properties of the material is indeed not valid in the HT phase. In this polymorph thermal fluctuations give rise to exceedingly large-amplitude oscillations of *J*_AB_ values and to nuclear configurations where the *J*_AB_ values within a given chain differ markedly from each other (see Fig. 5a). These observations raise the question whether it makes sense, from the physical point of view, to assign a single constant value to the intrachain *J*_AB_ in the HT phase of TTTA, even if the *static* approximation can furnish a reasonably good fitting to the measured data.

**Table 1:** Comparison between the experimental and computed magnetic susceptibility of the HT phase of TTTA at 300 K

	*χ* (emu mol^–1^)
Computed (static^*a*^)	6.7 × 10^–4^
Computed (vibrationally-averaged^*b*^)	5.2 × 10^–4^
Experimental	4.5 × 10^–4^

^*a*^Computed using the *J*_AB_ values associated with the *static* X-ray structure of HT at 300 K.

^*b*^Computed taking into account the thermal fluctuations at 300 K.

**Fig. 8:**
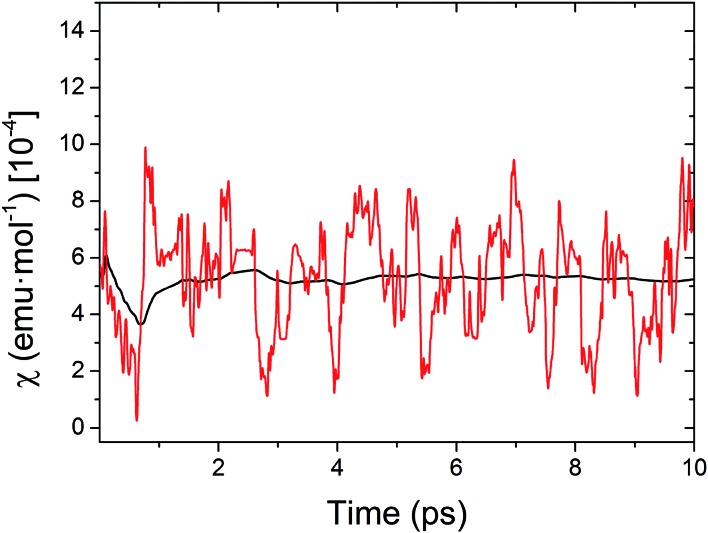
Time-resolved fluctuations of the sampled values of *χ* along the AIMD trajectory (red curve) and evolution of the computed cumulative running average of *χ* (black curve) for the HT phase of TTTA at 300 K.

At this point, it is important to stress that the direct physical observable is the magnetic susceptibility *χ*, instead of the *J*_AB_ values. Hence, the final step of our work was the evaluation of the vibrationally-averaged magnetic susceptibility, *χ̄*_vib_, of the HT polymorph at 300 K. *χ̄*_vib_ was calculated as the average of the *χ* value evaluated for all the nuclear configurations for which the *J*_AB_ values were monitored. The cumulative running average of the computed *χ* values achieves a well-converged finite value after *ca.* 5 ps of AIMD trajectory (Fig. 8). Remarkably, the value obtained for *χ̄*_vib_ is not only in very good agreement with the experimental data but it is also significantly lower (*ca.* 23%) than the *χ* value computed within the standard *static* approach, that is, the *χ* value obtained from the *J*_AB_ values calculated for the X-ray crystal structure (see Table 1). Notably important is that the *static* value of *χ* is in worse agreement with the measured data. It thus follows that the agreement between the computed and experimental *χ* is better when the thermal fluctuations are explicitly taken into consideration.

The small deviation between the computed *χ̄*_vib_ and experiment is most likely due to the fact that the one-dimensional magnetic model employed to compute *χ via* diagonalization of the Heisenberg Hamiltonian comprises only four spin centers (a larger magnetic model would thus be required to achieve a better agreement, see Fig. S12†). Note also that a tiny part of this small deviation might originate as well in the use of eqn (3) as an approximation to compute the *J*_AB_ values. That said, it should be stressed that, although *χ̄*_vib_ does not perfectly match the measured *χ*, our numerical analysis clearly demonstrates that thermal fluctuations play a prime role in defining the magnetic response of the HT phase of TTTA at 300 K. In the light of the results herein presented, we can easily understand why in our previous *static* study^19^ of the magnetism of TTTA we had to resort to the X-ray structure refined at 250 K to quantitatively reproduce the experimental value of *χ* at 300 K. As reflected in Table 1, the thermal fluctuations at 300 K result in a decrease in the value of *χ* with respect to the *static* susceptibility. In our previous work,^19^ where the nuclear motion was neglected, this decrease was effectively taken into consideration by using a crystal structure refined at a lower temperature, where the *J*_AB_ values between adjacent radicals are more antiferromagnetic (*J*_AB_ = –184 cm^–1^) than the corresponding *J*_AB_ values at 300 K (*J*_AB_ = –135 cm^–1^).

As commented on in the introduction, we have studied the interplay between thermal fluctuations and magnetism in TTTA only at 300 K. This is because our AIMD simulations describe the thermal motion of this material at this specific temperature and, thus, the subsequent analysis of the magnetic exchange interactions, and the vibrationally-averaged magnetic susceptibility (*vide infra*), is only valid to derive the value of *χ*(*T*) at 300 K. At this point, one can think about how the precedent analysis can be useful in the prediction of *χ*(*T*) values at different temperatures. In the present manuscript, it has been demonstrated that the key concept, in order to explain the magnetism of a material with large thermal fluctuations such as TTTA, is the statistical distribution of *J*_AB_ values at a given temperature. It thus follows that a change in this distribution must be ultimately responsible for an increase/decrease in the measured *χ*(*T*). For instance, it is known that the HT phase of TTTA features a value of *χ*(250 K) that is slightly lower than *χ*(300 K) (Fig. 2c). At 250 K, the vibrational motion of the molecules must be associated with thermal fluctuations of shorter amplitude than at 300 K, which means that, according to the analysis drawn in subsection 3, the value of 
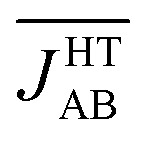
 would be less AFM. This, in turn, would translate into a larger value of *χ*(250 K), which is at odds with the experimental data. However, one should not forget the effect of the thermal contraction upon cooling, which entails smaller values of *d*_ip_ and *d*_sl_ for the average structural arrangement of adjacent radicals. Since smaller values of these variables are associated with more AFM *J*_AB_ values (see colored surfaces of Fig. 6), 
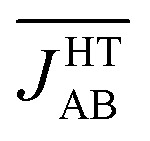
 will most likely be more AFM upon cooling even if the thermal fluctuations are less pronounced. This would explain the decrease in the *χ*(*T*) value of the HT phase of TTTA when cooling.

With reference to the LT phase of TTTA, the molecular motion at 300 K also results in large-amplitude oscillations of the *J*_AB_ values within the eclipsed dimers (see previous subsection 1). Yet the large majority of sampled *J*_AB_ values are so strongly antiferromagnetic that they lead to the same diamagnetic behavior predicted by the *static J*LT,X-rayAB value. Hence, in the particular case of the LT phase, the thermal fluctuations are not reflected in the experimentally measured *χ*.

## Conclusions

The analysis herein presented provides evidence that thermal fluctuations substantially affect the magnetic response of the HT phase of TTTA. In particular, our study brings to light a strong coupling between the electronic structure of this material and the vibrations of its constituent radicals. This coupling gives rise to large-amplitude oscillations of the magnetic exchange interactions, which, in turn, lead to a vibrationally-averaged magnetic susceptibility that differs substantially from the *static* susceptibility obtained using a “frozen” X-ray crystal structure, and that is in much better agreement with the experimental data. The discovery that the HT X-ray crystal structure of TTTA is not sufficiently representative to interpret the magnetic susceptibility of this material originates in the fact that the oscillations of key structural variables around their average values bring about strongly asymmetric variations of *J*_AB_.

Our findings go beyond the increasingly acknowledged fact that *J*_AB_ interactions feature a significant temperature-dependence in certain molecular systems due to thermal structural changes.^1–6^ Indeed, the huge asymmetric fluctuations of *J*_AB_ due to the nuclear motion in the HT phase of TTTA indicate that, at a given temperature, *J*_AB_ should not be treated as a constant value. Instead, one has to look at the statistical distribution of *J*_AB_ values in order to get a proper physical picture. Furthermore, the fluctuations observed for the *J*_AB_ values within a stack of the HT phase of TTTA have an important effect on the magnetic topology of the system. Specifically, our analysis demonstrates that the regular 1D chain topology that was previously proposed in order to interpret the magnetic properties of this phase does not properly reflect the physics of the system since, in many of the configurations sampled due to intermolecular vibrations, the *J*_AB_ values within a given chain differ considerably from each other.

Regarding the LT phase of TTTA, our simulations show that the vibrationally-averaged value of the *J*_AB_ between the radicals forming an eclipsed TTTA dimer is *ca.* 15% more antiferromagnetic than the *static* value obtained using the X-ray crystal structure. This means that thermal fluctuations have a non-negligible impact on the microscopic magnetic properties of LT, even if this phase does not feature any dynamic disorder. In this particular case, the impact of thermal fluctuations at the microscospic scale is not reflected at the macroscopic scale since both the *static J*_AB_ and the *J*_AB_ values that are sampled due to intermolecular vibrations are strongly antiferromagnetic and, therefore, both the *static* and *dynamic* approaches to the macroscopic magnetic properties predict a diamagnetic behavior. Yet, the results found for the LT phase are important because they suggest that thermal fluctuations can also play a notable role in defining the magnetic properties of ordered molecular crystals.

Overall, our work has important consequences in the field of molecular magnetism since it calls into question for the first time the standard common interpretation of the magnetic susceptibility based on *static* average structures. We do believe that the limitations of such a *static* approach, herein demonstrated for TTTA, are extensible to other molecule-based materials. Indeed, an improved *dynamic* perspective to describe magnetism should be adopted whenever the thermal fluctuations at a given temperature give rise to relative motions between spin carrying units or moieties that lead to pronounced non-linear (*e.g.* exponential) variations of the corresponding magnetic couplings. This might well be the case for organic radical magnets^41–45^ with dominant exchange interactions propagating through π–π labile networks, such as other members of the family of bistable or switchable dithiazolyl-based materials.^33–40^ In the context of transition metal complexes, the dynamic approach might be also required, for instance, in the “breathing” crystals of copper-nitroxide based molecular magnets family.^2^
