# Supplementary material for: Dynamical effects on the magnetic properties of dithiazolyl bistable materials
Source: Chem Sci. 2015 Jan 23;6(4):2371–81. doi: 10.1039/c4sc03930k (PMC5645919; doi:10.1039/c4sc03930k)
Supplement: Supplementary file 1 [file SC-006-C4SC03930K-s001.pdf]

Electronic Supplementary Material (ESI) for Chemical Science.
This journal is © The Royal Society of Chemistry 2015

Dynamical effects on the magnetic properties of dithiazolyl bistable materials

Sergi Vela[1], Mercè Deumal[1], Motoyuki Shiga[2], Juan J. Novoa[1], Jordi Ribas-Arino[1]*

[1] Departament de Química Física & IQTCUB, Facultat de Química, Universitat de Barcelona. Martí i Franquès 1, 08028,Barcelona, Spain

[2] Center for Computational Science and E-systems, Japan Atomic Energy Agency, 5-1-5, Kashiwanoha, Kashiwa, Chiba 277-8587, Japan

j.ribas@ub.edu

-Supporting Information-

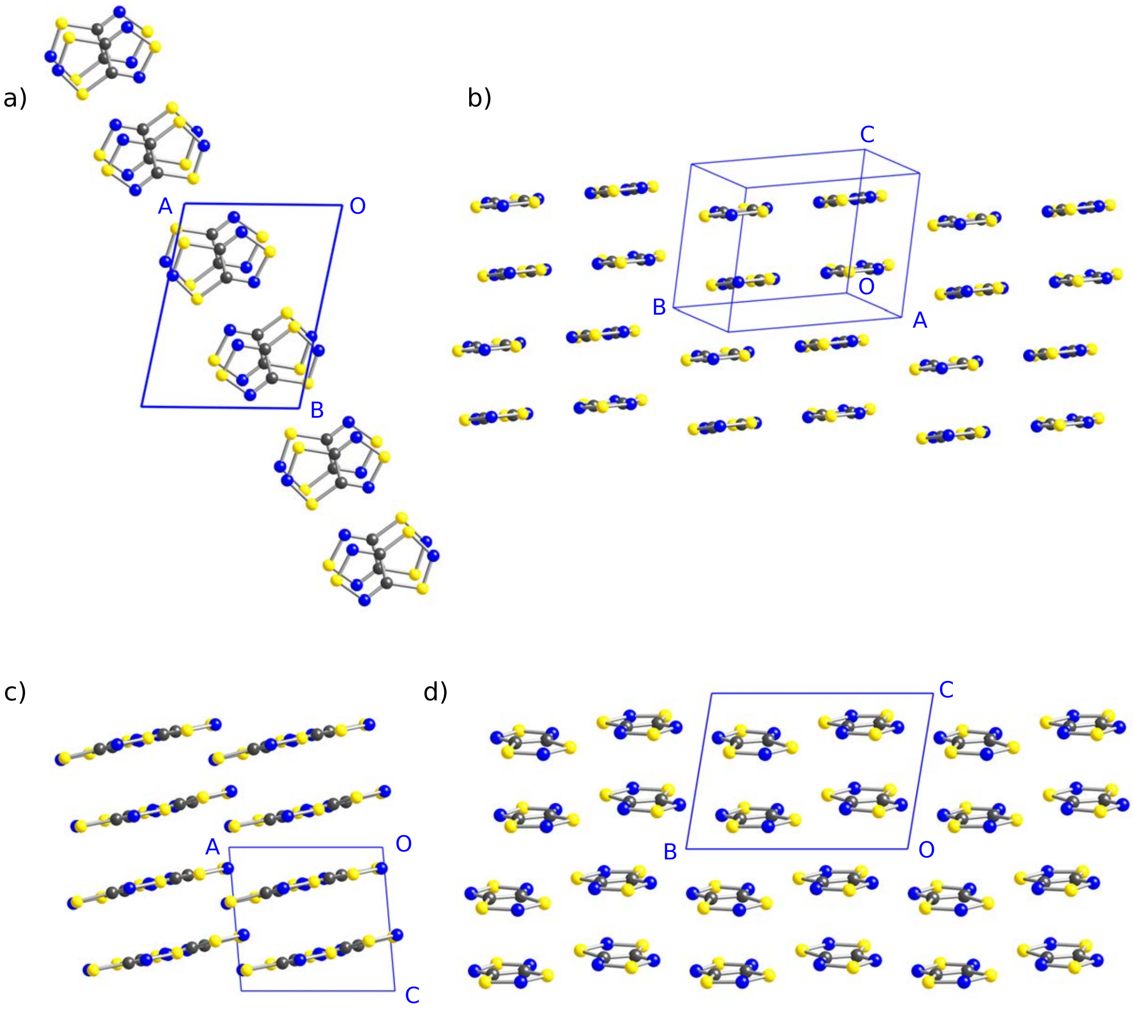

Figure S1: Different views of the crystal packing of the X-ray-resolved LT polymorph of TTTA at 300 K (CCDC refcode = SAXPOW06). This triclinic crystal comprises 2D layers formed by π-stacks of TTTA radicals that are laterally linked by a series of intermolecular S···S contacts and six-center S···N contacts (see Figure S3). The different 2D layers of the LT polymorph are, in turn, linked by a series of interstack S···N and S···S contacts (see Figure S4). The 2D layers in LT contain one single molecular plane orientation. For a given 2D layer, the figure shows: (a) a plane of TTTA radicals perpendicular to the π stacks, viewed along *c*; (b) lateral view of the plane displayed in (a) to show the single molecular plane orientation of TTTAs. For a set of 2D layers, the figure shows: (c) π stacks viewed along *b*; (d) π stacks viewed along *a*.

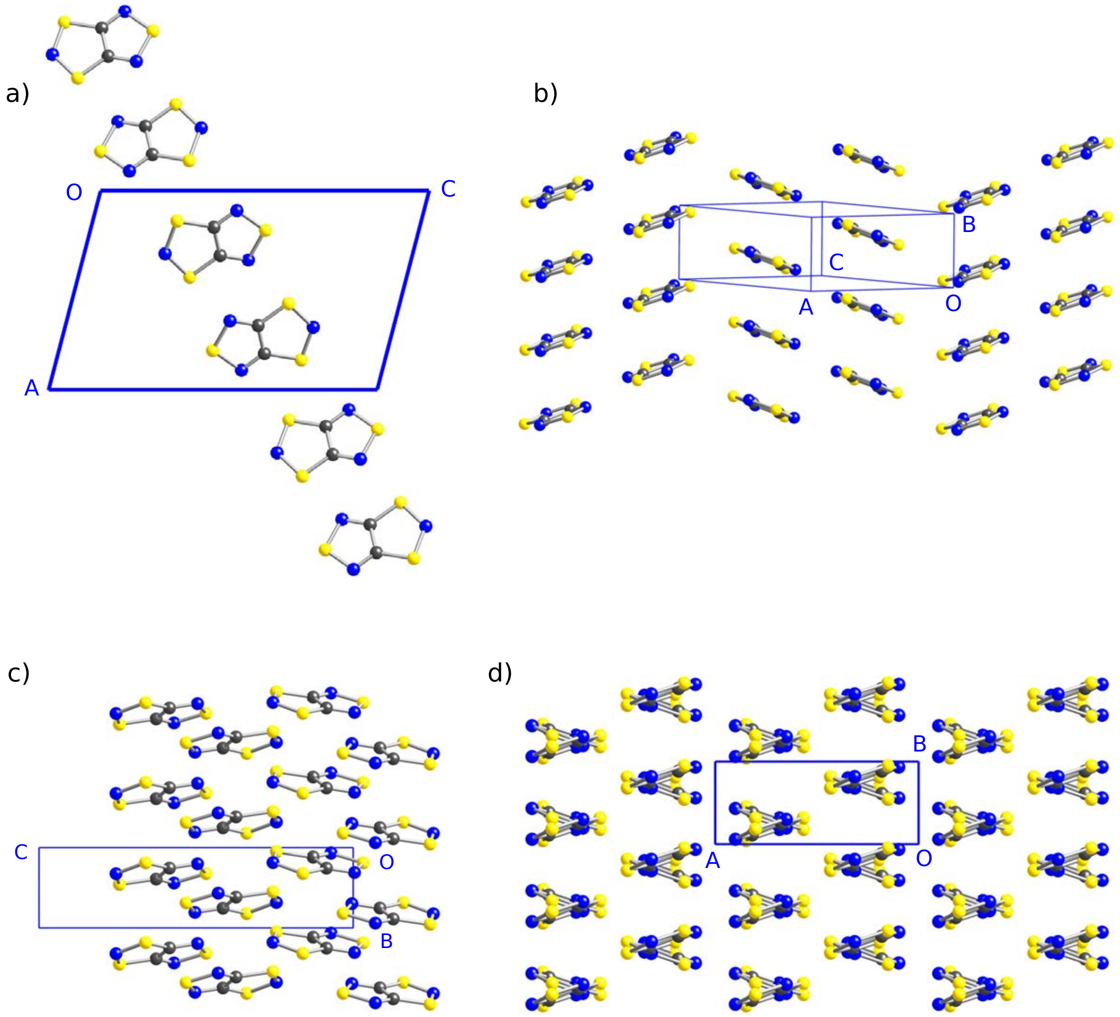

Figure S2: Different views of the crystal packing of the X-ray-resolved HT polymorph of TTTA at 300 K (CCDC refcode = SAXPOW05).). This monoclinic crystal comprises 2D layers formed by π-stacks of TTTA radicals that are laterally linked by a series of intermolecular S···S contacts and six-center S···N contacts (see Figure S3). The different 2D layers of the LT polymorph are, in turn, linked by a series of interstack S···N and S···S contacts (see Figure S4). The 2D layers in LT contain two different molecular plane orientations. For a given 2D layer, the Figure shows: (a) a plane of TTTA radicals perpendicular to the π stacks, viewed along *b*; (b) lateral view of the plane displayed in (a). For a set of 2D layers, the Figure shows: (c) π stacks viewed along *a*; (d) π stacks viewed along *c*.

a)

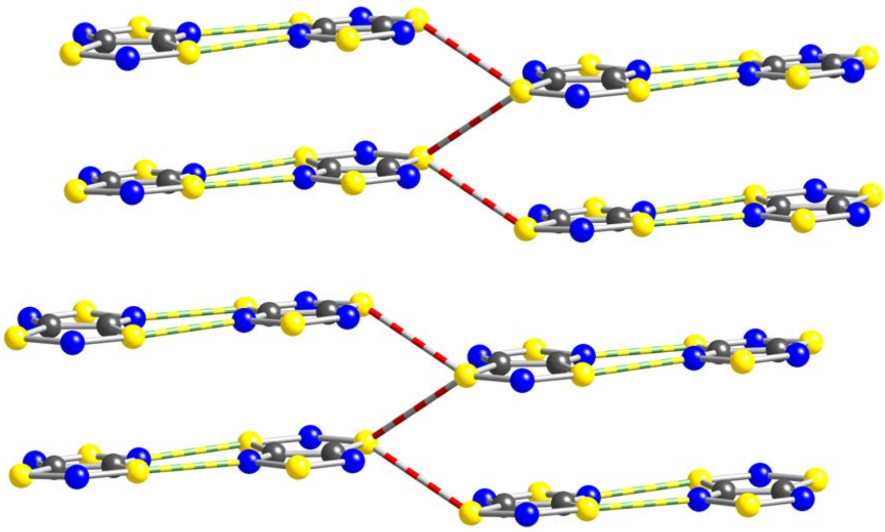

b)

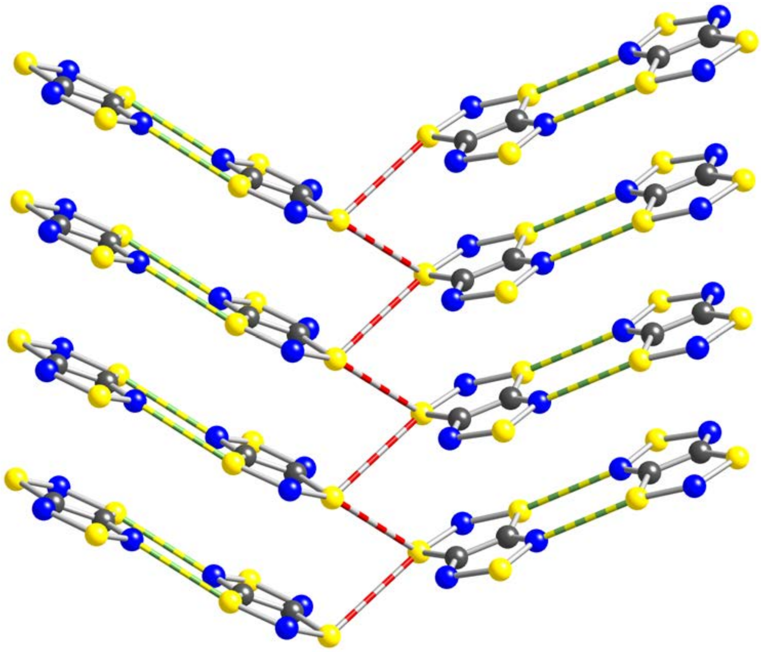

Figure S3: For a given 2D layer, stacking of radicals in the X-ray resolved LT-300 (a) and HT-300 (b) structures. The grey and red cylinders mark the lateral S···S contacts of TTTA molecules between neighboring stacks; the green and yellow cylinders identify the lateral six-center N···S contacts. The two types of lateral contacts that are highlighted are those associated with distances shorther than the sum of the corresponding van der Waals radii.

a)

b)

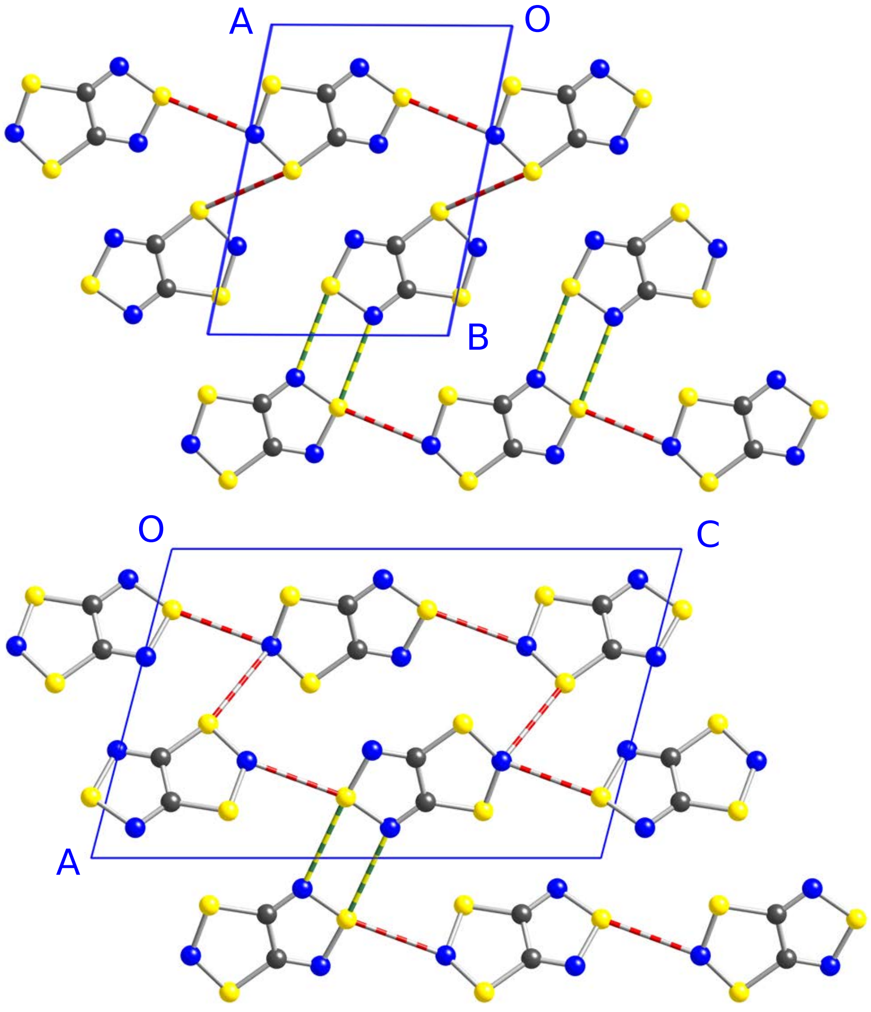

Figure S4: For a set of 2D layers, top view of the stacks of TTTA radicals in the X-ray resolved LT-300 (a) and HT-300 (b) structures. The grey and red cylinders mark the lateral S···N or S···S contacts between neighboring TTTA molecules; the green and yellow cylinders identify the lateral four-center N···S contacts. The only lateral contacts that are highlighted are those associated with distances shorther than the sum of the corresponding van der Waals radii.

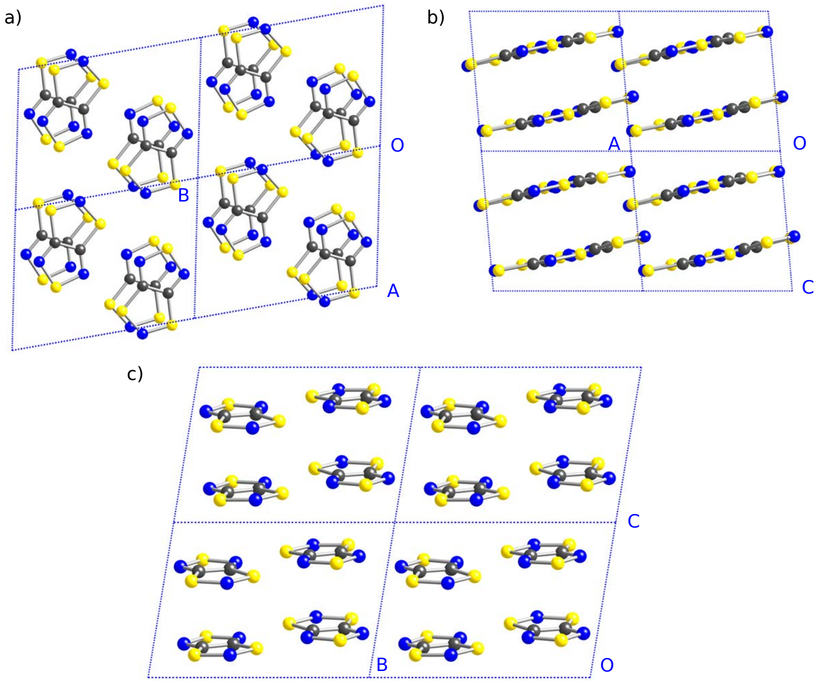

Figure S5: Three different views of the supercell used in the AIMD simulations of the LT polymorph of TTTA. The supercell for LT has been constructed by duplicating the unit cell in all three spatial directions. The lattice parameters of this supercell are collected in Table S1.

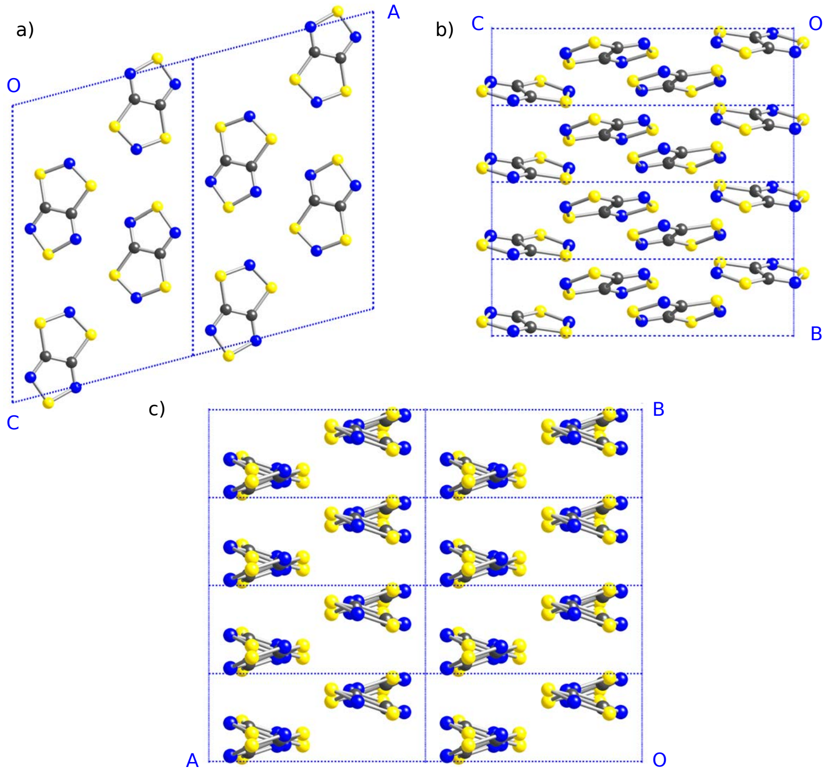

Figure S6: Three different views of the supercell used in the AIMD simulations of the HT polymorph of TTTA. The supercell for HT has been constructed by replicating the unit cell in two of the spatial directions. Specifically, the relations between the supercell (sc) vectors and the unit cell (uc) vectors are: $a_{sc} = 2{\cdot}a_{uc}$; $b_{sc} = 4{\cdot}b_{uc}$; $c_{sc} = c_{uc}$. The lattice parameters of this supercell are collected in Table S1.

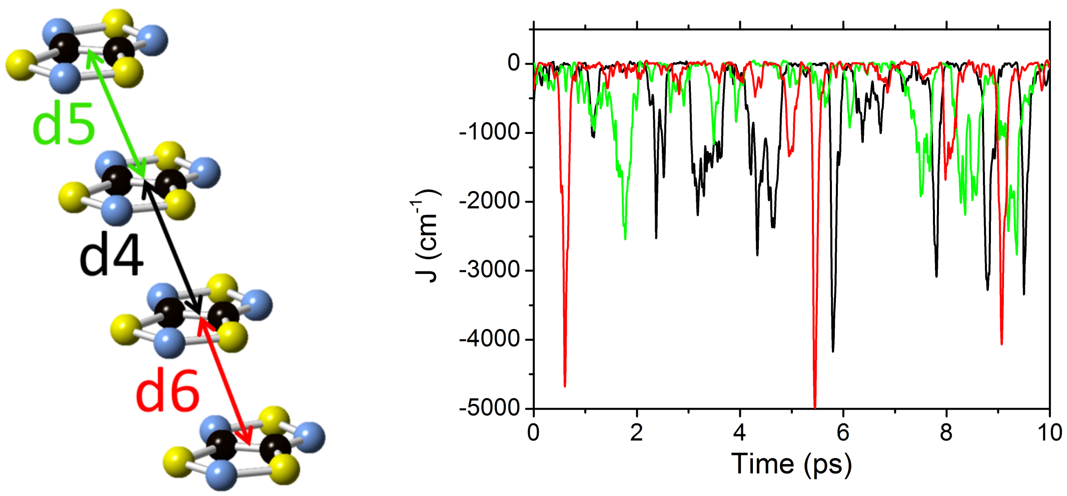

Figure S7. Time-resolved fluctuations of the J_{AB}'s for dimers 4-6 of the HT phase.

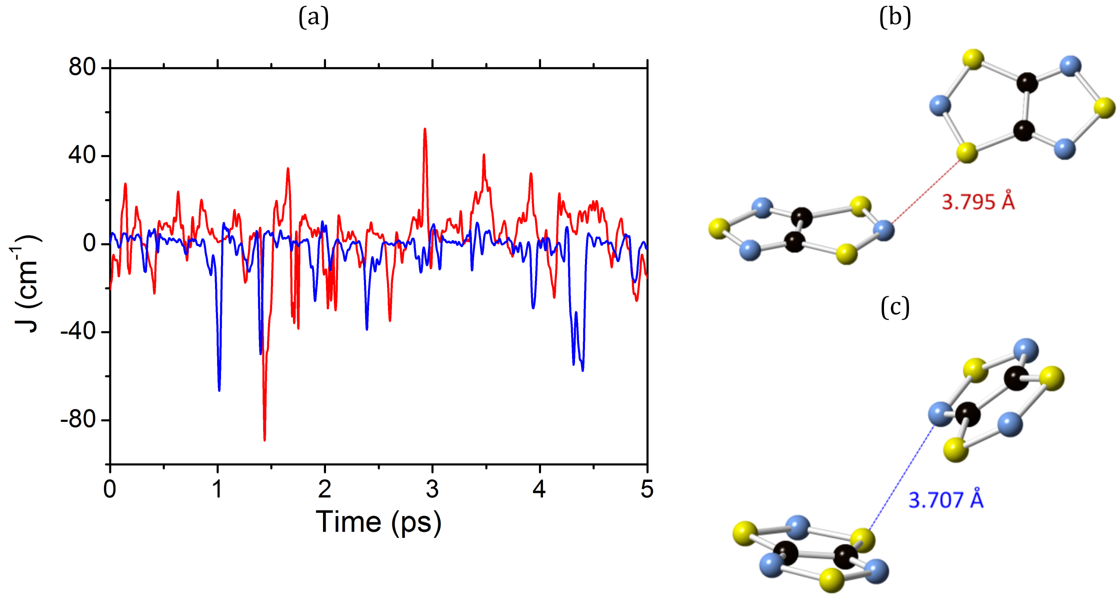

Figure S8. (a) J_{AB} time-resolved fluctuations for the interstack interactions corresponding to (b) dimer 7 (red curve) and (c) dimer 8 (blue curve) of the HT phase. Among all the possible interstack radical pairs, only the two pairs displayed in b) and c) have been selected because these were previously shown[1] to be the interstack dimers associated with a larger J_{AB} value.

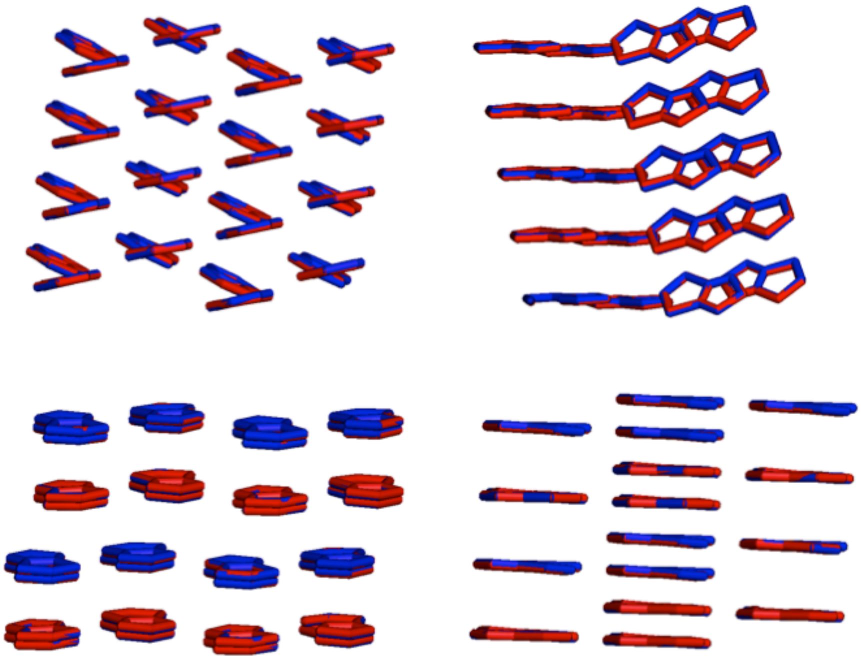

Figure S9: Lateral views of the HT-300 polymorph (*top*) and the LT-300 polymorph of TTTA (*bottom*). The average structures obtained from the AIMD simulations (depicted in red) are superimposed on the respective X-ray crystal structures (depicted in blue).

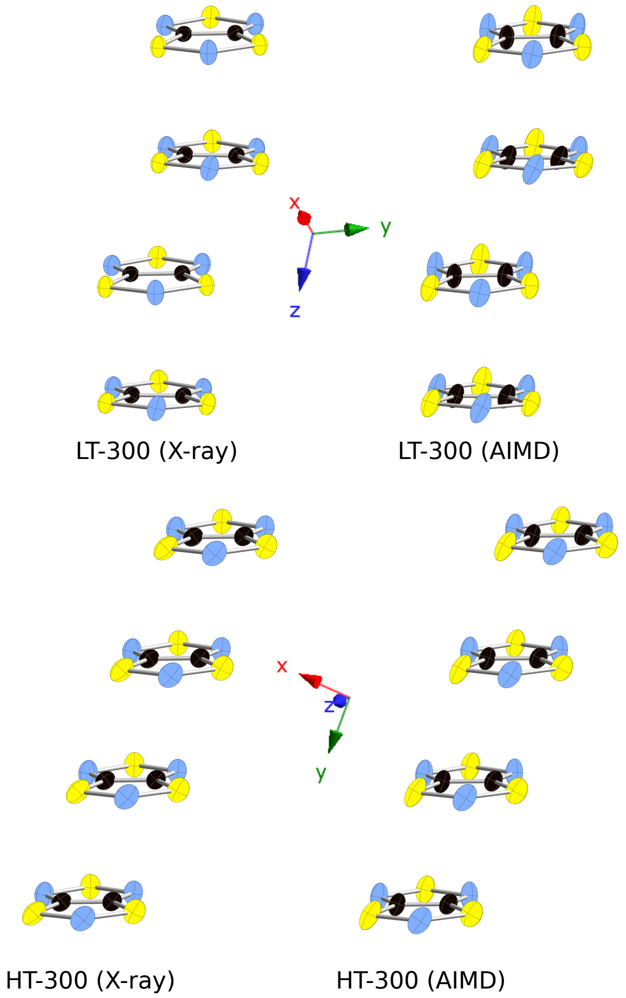

Figure S10: Thermal ellipsoids of the atoms of the TTTA molecules in the LT-300 and HT-300 phases. *Left*: experimental thermal ellipsoids obtained from X-ray measurements; *Right*: thermal ellipsoids obtained from the ab initio molecular dynamics (AIMD) simulations. The displayed ellipsoids correspond to a probability of 50%.

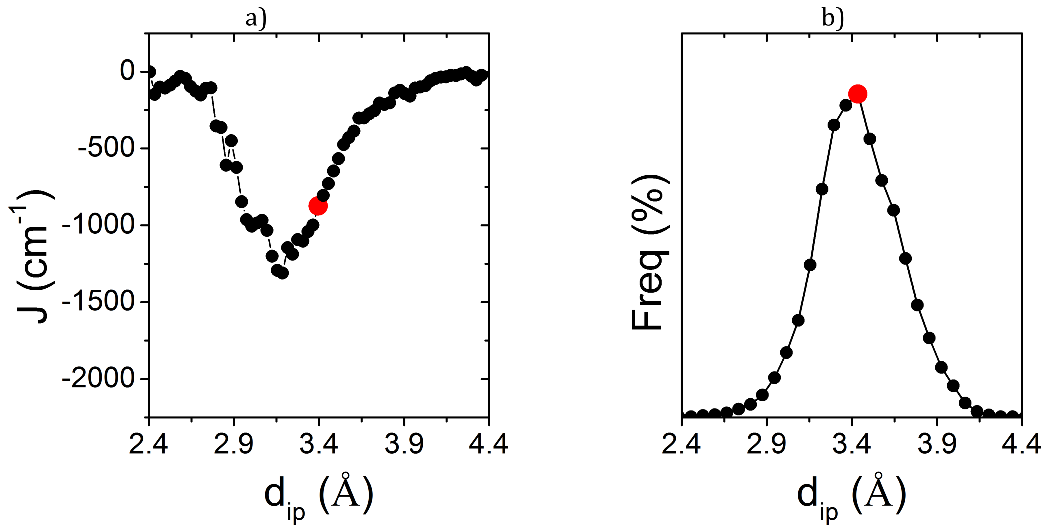

Figure S11. (a) Dependence of the J_{AB} value between adjacent radicals on their interplanar distance (d_{ip}) and (b) the probability distribution function (PDF) associated with d_{ip}. Each J_{AB} value on (a) has been computed as an average of all the computed J_{AB}'s for all the configurations sampled for a given value of d_{ip}. The red mark indicates the value of d_{ip} at the HT X-ray structure. Note that d_{ip} is defined in Figure 5 of the main text.

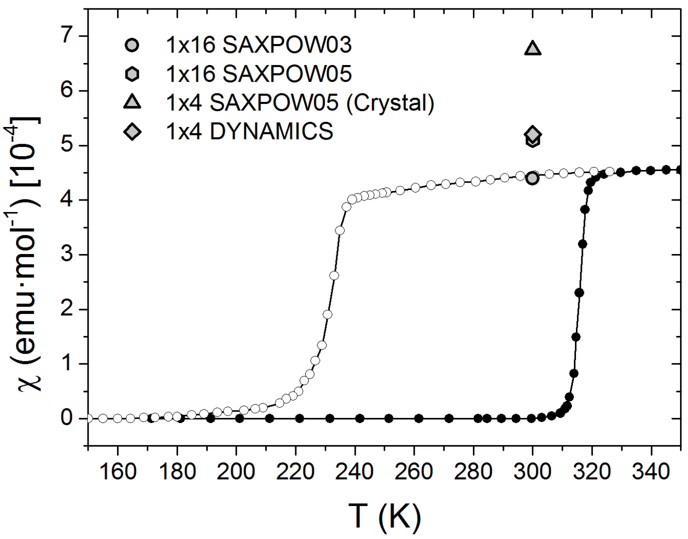

Figure S12. Comparison of the experimental magnetic susceptibility curves and the values obtained by simulation. The experimental χ(T) curves for both phases of TTTA within the bistability range of temperatures are displayed in filled circles (heating mode) and empty circles (cooling mode). The computed χ values displayed at 300 K are the following ones: i) *static* χ value computed for the crystal structure refined at 250 K using a 1D magnetic model system with 16 spin centers (1x16 SAXPOW03); ii) *static* χ value computed for the crystal structure refined at 300 K using a 1D magnetic model system with 16 spin centers (1x16 SAXPOW05); iii) *static* χ value computed for the crystal structure refined at 300 K using a 1D magnetic model system with 4 spin centers (1x4 SAXPOW05); iv) vibrationally-averaged magnetic susceptibility computed with a 1D magnetic model system with 4 spin centers (1x4 DYNAMICS). Note that the experimental data have been taken from Ref. 1. The results collected in this Figure demonstrate that the computed *static* magnetic susceptibility using the J_{AB} values of the X-ray crystal structure of HT at 300 K depends on the number of spin centers included in the one-dimensional magnetic model systems. In particular, it is observed that the use of a 1D-model with 16 spin centers (hexagon marker), instead of a model with 4 spin centers (triangle marker), leads to a better agreement with the experimental data. This suggests that if $\bar{\chi}_{\text{vib}}$ had been computed using magnetic models of 16 spin centers, our results would be even closer to the experimental value of χ at 300K. However, an expansion of our model up to 16 spins would have a prohibitive computational cost: a new AIMD run of an extended super cell, hosting several stacks of 16 TTTA molecules each, would be a necessary step previous to the computation of the J_{AB} values of 15 dimers along ten picoseconds (i.e. 150000 new J_{AB} calculations).

Table S1: Lattice parameters of the supercells employed in the calculations of the LT and HT polymorphs of TTTA.[1]

	a	*b*	*c*	α	β	γ
LT[2]	15.062	20.046	14.048	100.598	96.978	77.638
HT	18.888	14.844	15.063	90.0	104.628	90.0

[1] The *a*, *b* and *c* parameters are given in Angstrom.

[2] The (*a*,*b*,*c*) supercell parameters of LT and HT are multiples of the (*a*,*b*,*c*) parameters defining the unit cells of the LT-300 and HT-300 X-ray resolved structures, respectively. The relations between the supercell (sc) parameters and the unit cell (uc) parameters are the following ones: $a_{LT,sc} = 2 \cdot a_{LT,uc}$; $b_{LT,sc} = 2 \cdot b_{LT,uc}$; $c_{LT,sc} = 2 \cdot c_{LT,uc}$; $a_{HT,sc} = 2 \cdot a_{HT,uc}$; $b_{HT,sc} = 4 \cdot b_{HT,uc}$; $c_{HT,sc} = c_{HT,uc}$

Table S2: Computed J_{AB} values (given in cm^{-1}) for an eclipsed dimer of the LT polymorph and for a pair of adjacent radicals within a stack of the HT polymorph of TTTA. The J_{AB} values calculated with two different electronic structure methods are compared.

	**LT**[a]	**HT**[b]
UB3LYP/6-31+G(d)[c]	-1755	-135
MRMBMP2/Aug-cc-pVDZ[d]	-2109	-107

[a] The coordinates of the eclipsed dimer (see Figure 2a in the main text) were extracted from the X-ray crystal structure of LT recorded at 300 K.

[b] The coordinates of the pair of adjacent radicals (see Figure 2b in the main text) were extracted from the X-ray crystal structure of HT recorded at 300 K.

[c] These J_{AB} values were obtained using Equation 3 of the main text.

[d] These are *ab initio* multireference many-body second-order perturbation (MRMBPT2) calculations[2] using a 10 orbital and 10 electron active space. These calculations were performed using GAMESS-US[3] program with an Aug-cc-pVDZ basis set[4]. Note that at this level of calculation, the singlet state is an eigenstate of $\hat{S}^2$ (*i.e.*, it is not spin-contaminated like in the broken symmetry approximation used in the DFT calculations) and thus $J_{AB} = (E^S - E^T)/2$.

Table S3. Average J_{AB} values for each of the *d1-d6* dimers of the HT phase and *d1-d2* dimers of the LT phase. See also the average value and error for the collection of all J_{AB} values evaluated for each crystal phase. The J_{AB} values obtained from the radical pairs of the static X-ray crystal structure are collected at the bottom of the table. Remarkably, the average J_{AB} for every pair of radicals is much more antiferromagnetic (especially in the case of the HT phase) than the J_{AB} value obtained from the *static* X-ray crystal structure.

	$\bar{J}_i^{HT}$ **(cm⁻¹)**	$\bar{J}_i^{LT}$ **(cm⁻¹)**
Dimer #1	−397,9	−1947,0
Dimer #2	−394,0	−2089,6
Dimer #3	−604,1	
Dimer #4	−522,9	
Dimer #5	−415,6	
Dimer #6	−283,7	
AVERAGE	$\bar{J}_{AB}^{HT} = -436{,}4$	$\bar{J}_{AB}^{LT} = -2018{,}3$
Error	±40	±35
	$J_{AB}^{HT,X-ray} = -135{,}6$	$J_{AB}^{LT,X-ray} = -1755{,}0$

References

[1] Clarke, C.S.; Jornet-Somoza, J.; Mota, F.; Novoa, J.J.; Deumal, M. *J. Am. Chem. Soc.* **2010**, *132*, 17817.

[2] (a) Wolinski, K.; Sellers, H. L.; Pulay, P. *Chem. Phys. Lett.* **1987**, *140*, 225; (b) Hirao, K. *Int. J. Quantum Chem. Symp.* **1992**, *26*, 517; (c) Nakano, H. *J. Chem. Phys.* **1993**, *99*, 7983.

[3] Schmidt, M. W.; Baldridge, K. K.; Boatz, J. A.; Elbert, S. T.; Gordon, M. S.; Jensen, J. H.; Kosecki, S.; Matsunaga, N.; Nguyen, K. A.; Su, S. J.; Windus, T. L.; Dupuis, M.; Montgomery, J. A. *J. Comput. Chem.* **1993**, *14*, 1347.

[4] (a) Dunning, Jr., T. H. *J. Chem. Phys.* **1989**, *90*, 1007; (b) Kendall, R. A.; Dunning, Jr., T. H.; Harrison, R. J. *J. Chem. Phys.* **1992**, *96*, 6796; (c) Woon, D. E.; Dunning, Jr., T. H. *J. Chem. Phys.* **1993**, *98*, 1358.
